# Integrated Transcriptomic and Metabolomic Analysis Reveals the Molecular Regulatory Mechanism of Flavonoid Biosynthesis in Maize Roots under Lead Stress

**DOI:** 10.3390/ijms25116050

**Published:** 2024-05-31

**Authors:** Zhaolai Guo, Xinqi Yuan, Ting Li, Sichen Wang, Yadong Yu, Chang’e Liu, Changqun Duan

**Affiliations:** 1 Yunnan Key Laboratory of Plateau Ecology and Degraded Environment Restoration, School of Ecology and Environmental Science, Yunnan University, Kunming 650091, China; 18314522882@163.com (Z.G.); xqyuan2022@163.com (X.Y.); litingkm1891@163.com (T.L.); wangsichen0101@163.com (S.W.); 13685311866@163.com (Y.Y.); change@ynu.edu.cn (C.L.); 2 Yunnan Provincial Innovative Research Team of Environmental Pollution, Food Safety, and Human Health, Institute of Environmental Remediation and Human Health, School of Ecology and Environment, Southwest Forestry University, Kunming 650224, China

**Keywords:** lead, maize root, flavonoids, transcriptomics, metabolomics

## Abstract

Flavonoids are secondary metabolites that play important roles in the resistance of plants to abiotic stress. Despite the widely reported adverse effects of lead (Pb) contamination on maize, the effects of Pb on the biosynthetic processes of flavonoids in maize roots are still unknown. In the present work, we employed a combination of multi-omics and conventional assay methods to investigate the effects of two concentrations of Pb (40 and 250 mg/kg) on flavonoid biosynthesis in maize roots and the associated molecular regulatory mechanisms. Analysis using conventional assays revealed that 40 and 250 mg/kg Pb exposure increased the lead content of maize root to 0.67 ± 0.18 mg/kg and 3.09 ± 0.02 mg/kg, respectively, but they did not result in significant changes in maize root length. The multi-omics results suggested that exposure to 40 mg/kg of Pb caused differential expression of 33 genes and 34 metabolites related to flavonoids in the maize root system, while 250 mg/kg of Pb caused differential expression of 34 genes and 31 metabolites. Not only did these differentially expressed genes and metabolites participate in transferase activity, anthocyanin-containing compound biosynthetic processes, metal ion binding, hydroxyl group binding, cinnamoyl transferase activity, hydroxycinnamoyl transferase activity, and flavanone 4-reductase activity but they were also significantly enriched in the flavonoid, isoflavonoid, flavone, and flavonol biosynthesis pathways. These results show that Pb is involved in the regulation of maize root growth by interfering with the biosynthesis of flavonoids in the maize root system. The results of this study will enable the elucidation of the mechanisms of the effects of lead on maize root systems.

## 1. Introduction

Flavonoids comprise a class of polyphenolic plant secondary metabolites that contain the phenylchromone ring skeleton (C6-C3-C6) as the basic component [1]. There are more than 9000 known flavonoids, which are compounds classified as flavones, flavonols, isoflavones, anthocyanidins, and proanthocyanidins [2,3,4,5,6]. Flavonoids are generally found in plant organs (e.g., roots, stems, and leaves). Most flavonoids combine with sugars to form glycosides or carbon sugar groups in plants, whereas a small portion exists in the free state [7,8]. Due to their unique chemical structures, flavonoids have multiple functions, including strong antioxidant activity, regulating leaf stomatal closure, and complexing with heavy metal elements [9,10]. Abiotic stress can disrupt the pathways of flavonoid biosynthesis in plants, resulting in interruption of the synthesis of various flavonoids (e.g., flavonoids, isoflavonoids, and anthocyanins), leading to dysfunction/loss of function and ultimately affecting the plant’s ability to resist stress [11,12,13,14,15]. Considering the important functions of flavonoids, their synthesis pathways under abiotic stress have received significant attention.

Lead (Pb) is the second-most toxic metallic element in nature. Anthropogenic activities have led to the accumulation of large quantities of Pb in the soil environment, potentially threatening environmental safety and human health [16]. Maize is an economically important crop, and many maize crops are currently polluted by heavy metals. Owing to its well-developed root system and strong metal tolerance, maize is often used to evaluate the toxic effects of and adaptation mechanisms to heavy metals in plants [17]. Studies have demonstrated that exposure to Pb (40–2550 mg/kg) for 14–60 days can lead to a series of toxic effects in maize, including oxidative damage [18], retarding the development and growth of roots, stems, and leaves [19], disrupting the integrity of cell membranes, regulating the expression of ATP-binding cassette transporter, and causing stomatal closure [20,21]. Despite that the adverse effects of Pb on maize have been widely reported, the effects of Pb on flavonoid biosynthesis in maize roots and the associated molecular regulatory mechanisms remain unknown. The effects of toxicity in maize induced by heavy metal are often closely related to the regulation of flavonoids [22]. Studies have shown that an increase in the content of flavonoids can eliminate reactive oxygen species and help plants resist plant oxidative damage [10,23]. Other studies have found that flavonoids can bind to heavy metals, thus reducing the levels of free metal elements in cells and ultimately reducing the toxic effects of heavy metals on plants [24,25,26,27,28]. These studies suggest that studying the biosynthetic processes and molecular regulatory mechanisms of flavonoids is significant for a deeper understanding of the physicochemical changes in plants under heavy metal stress.

In recent years, multi-omics research has provided systems biology with a new approach to studying gene expression and metabolite accumulation mechanisms related to environmental stress [29,30]. Their high throughput and high sensitivity have led to the wide application of transcriptomics and metabolomics to evaluating the effects of abiotic stress on plants [31]. Transcriptomics can be used to identify changes in global gene expression and to investigate the mechanisms of heavy metal toxic effects [32]. Metabolomics can be used to systematically reveal the effects of heavy metals on metabolic pathways and help identify biomarkers for heavy metals [33]. Specifically, recently developed techniques for joint multi-omics analyses can better enable a comprehensive and deterministic assessment of biohazardous effects and mechanisms under abiotic stress than single-omics can [34]. Therefore, it should be possible to evaluate and clarify the effects of Pb on the biosynthesis of maize flavonoids and their regulatory molecular mechanisms by combining multi-omics and traditional techniques.

The primary objective of this work was to reveal, by integrating transcriptomics and metabolomics, the effect of Pb on the biosynthesis of flavonoids and the underlying molecular regulatory mechanisms in maize roots. An additional goal was to determine the involvement of flavonoid compounds in the underlying mechanism of Pb-induced changes in maize root phenotypes. Several physiological and biochemical indices of maize roots, including root morphology, metal content, and root length, were analyzed in this study.

## 2. Results

### 2.1. Pb Accumulation in Maize Roots

**Figure 1:**
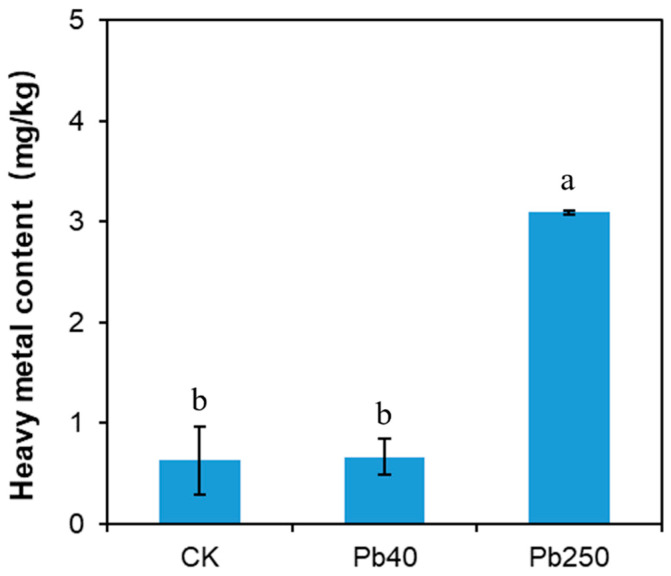
Accumulation of Pb in maize roots under different Pb concentration treatments. Different letters represent significant differences in the Pb treatment group compared to the control group (*p* < 0.05). CK refers to the control group. Pb40 and Pb250 refer to the concentrations of lead at 40 mg/kg and 250 mg/kg, respectively.

The results showed that high levels of Pb exposure caused significant accumulation of Pb in maize roots (Figure 1). Specifically, 40 mg/kg Pb exposure increased the Pb concentration to 0.67 ± 0.18 mg/kg, while 250 mg/kg Pb exposure led to a significant increase to 3.09 ± 0.02 mg/kg. The 250 mg/kg Pb exposure resulted in a fourfold higher Pb accumulation in maize roots than the 40 mg/kg Pb exposure.

### 2.2. Phenotypic Characterization of Maize Roots

**Figure 2:**
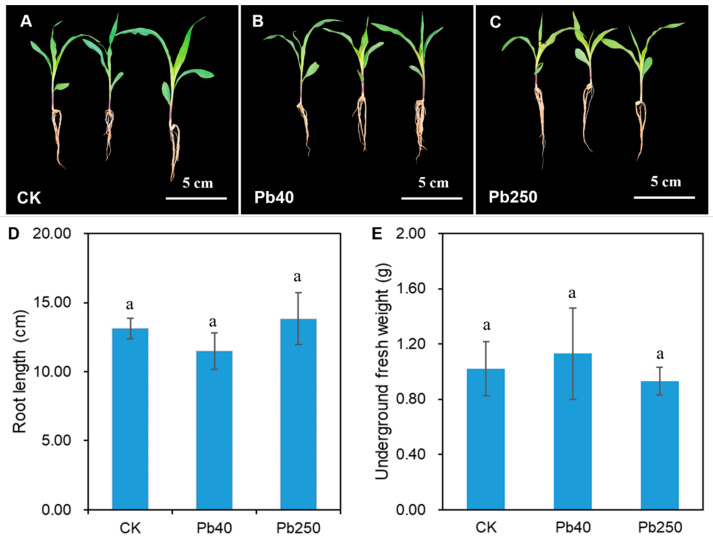
Phenotypic analysis of maize roots under Pb stress. (**A**–**C**) Phenotypic maps of maize roots in different treatment groups. (**D**) The root length of maize roots in different treatment groups. (**E**) Underground fresh weight of maize roots in different treatment groups. Pb40 and Pb250 refer to the concentrations of lead at 40 mg/kg and 250 mg/kg, respectively. In Figure (**D**,**E**), the letters represent significant differences between different treatments.

The morphological characteristics of maize roots are shown in Figure 2. The results indicated that the maize root system was multi-rooted. Under the control treatment, the maize roots had several whisker roots and did not show any breakage. Under the 40 mg/kg Pb treatment, the maize roots had more developed whisker roots, but the root system was shorter. The 250 mg/kg Pb treatment resulted in no significant change in the whisker roots, and the individual roots had different lengths. There were no statistically significant differences in the length or fresh weight of maize roots following Pb exposure. Compared with the control treatment, the 40 mg/kg Pb treatment caused a decrease in the length of maize roots (11.50 ± 1.32 cm), resulting in a slight increase in their fresh weight (1.13 ± 0.33 g). Despite a slight decrease in the fresh weight of maize roots (0.93 ± 0.10 g), the 250 mg/kg Pb treatment did not significantly affect maize root length (13.83 ± 1.89 cm).

### 2.3. Transcriptomic Analysis of Maize Root Flavonoids

**Figure 3:**
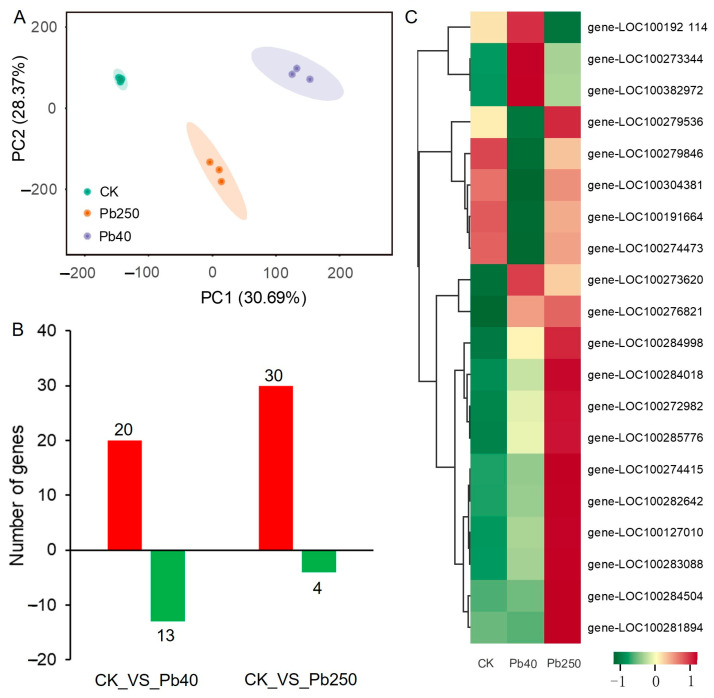
Differentially expressed genes related to flavonoids in maize roots following Pb exposure. (**A**) Plot of the PCA scores among the different treatment groups; (**B**) differentially expressed genes among the different treatment groups; (**C**) hierarchical cluster analysis of gene expression. Pb40 and Pb250 refer to the concentrations of lead at 40 mg/kg and 250 mg/kg, respectively.

The results of transcriptomic analysis showed that Pb exposure led to a large increase in the number of differentially expressed flavonoid genes (Figure 3). Principal component analysis (PCA) (Figure 3A) showed that the first component accounted for 30.69% of the variance and the second component for 28.37%, indicating the separation of the transcriptomes of the Pb-treated groups and the control group. The 40 mg/kg Pb treatment resulted in differential expression of 33 flavonoid-related genes, of which 20 were upregulated and 13 were downregulated (Figure 3B). The 250 mg/kg Pb treatment resulted in the differential expression of 34 flavonoid-related genes, of which 30 were upregulated and four were downregulated (Figure 3B). The information for the Pb-induced flavonoid genes is listed in Tables S2 and S3.

The clustering heat map results (Figure 3C) showed that the 40 mg/kg Pb treatment upregulated the flavonoid genes LOC100273344 (putative cytochrome P450 superfamily protein), LOC100192114 (anthocyanidin 3-O-glucoside 2″-O xylosyl transferase), LOC100382972 (coumarin hydroxycinnamoyl transferase), LOC100273620 (jasmone-regulated gene 21), and LOC100276821 (chalcone flavanone isomerase 1) (Figure 3C). The treatment downregulated LOC100279536 (putative cytochrome P450 superfamily protein), LOC100276821 (chalcone flavanone isomerase 1), LOC100279536 (cytochrome P450 superfamily protein), LOC100279846 (UDP-glycosyltransferase 88A1), LOC100274381 (ubiquitin ligase E2-ribosomal protein pseudogene), LOC100191664 (UDP-glycosyltransferase 88A1), and LOC100274473 (putative cytochrome P450 superfamily protein) (Figure 3C). The 250 mg/kg Pb treatment upregulated the flavonoid genes LOC100279536 (putative cytochrome P450 superfamily protein), LOC100284998 (trans-cinnamate 4-monooxygenase), LOC100284018 (chalcone–flavanone isomerase), LOC100272982 (anthocyaninless 4), LOC100285776 (leucoanthocyanidin dioxygenase), LOC100274415 (chalcone synthase), LOC100282642 (chalcone synthase), LOC100127010 (anthocyaninless 2), LOC100283088 (anthocyanidin 5,3-O-glucosyltransferase), LOC100284504 (dihydroflavonol-4-reductase), and LOC100281894 (transferase), as well as downregulated the genes LOC100192114 (anthocyanidin 3-O-glucoside 2″-O-xylosyltransferase) and LOC100191664 (UDP-glycosyltransferase 88A1). To verify the reliability of the transcriptional gene expression results, five differentially expressed genes were randomly selected for qPCR analysis. qPCR revealed (Table 1) that the transcript levels of the LOC100286107, LOC100275381, LOC100285776, LOC542712, and LOC100192114 genes were basically consistent with the gene expression results. This indicated reliable results for the differentially expressed genes in maize roots under exposure to different concentrations of Pb.

Gene Ontology (GO)-based functional analyses (Table 2) showed that the 40 and 250 mg/kg Pb treatments induced differentially expressed genes involved in flavonoid biosynthesis (GO:0009813), transferase activity (GO:0016758), iron ion binding (GO:0005506), anthocyanin-containing compound biosynthetic process (GO:0009718), metal ion binding (GO:0046872), hydroxycinnamoyl transferase activity (GO:0050734), flavanone 4-reductase activity (GO:0047890), chalcone isomerase activity (GO:0045430), flavanone 4-reductase activity (GO:0047890), dihydrokaempferol 4-reductase activity (GO:0045552), isoflavone 2′-hydroxylase activity (GO:0033773), and naringenin–chalcone synthase activity (GO:0016210).

### 2.4. Metabolomic Analysis of Maize Roots

**Figure 4:**
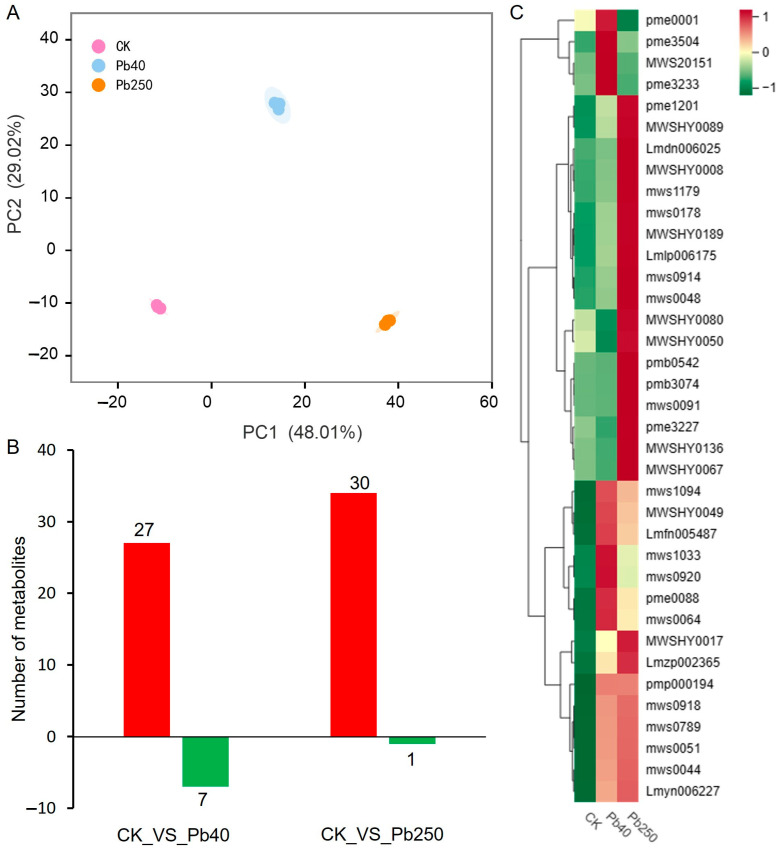
Analysis of differentially expressed metabolites of flavonoids in maize roots following Pb exposure. (**A**) Plot of the PCA scores among different treatment groups; (**B**) differentially expressed metabolites among different treatment groups; (**C**) hierarchical cluster analysis of metabolite expression. Pb40 and Pb250 refer to the concentrations of lead at 40 mg/kg and 250 mg/kg, respectively.

The metabolomic results demonstrated that Pb exposure resulted in a large number of differentially expressed flavonoid metabolites (Figure 4). PCA showed that the first and second components accounted for 48.01 and 29.02% of the variance, indicating good differentiation among the treatment groups (Figure 4A). The 40 mg/kg Pb treatment led to differential expression of 34 flavonoid-related metabolites in maize roots, of which 27 were upregulated and 7 downregulated (Figure 4B). The 250 mg/kg Pb treatment led to differential expression of 31 flavonoid-related metabolites, of which 30 were upregulated and 1 was downregulated (Figure 4B). The details of these metabolites are provided in Tables S4 and S5.

The heat map (Figure 4C) revealed that 40 mg/kg Pb upregulated the flavonoid metabolites pme0001 (hesperetin-7-O-neohesperidoside (neohesperidin)), pme3504 (formononetin-7-O-glycoside (ononin)), pme3233 (calycosin), mws20151 (apigenin), mws1094 (aromadendrin (dihydrokaempferol)), mws1033 (homoeriodictyol), and mws0920 (tricetin (5,7,3′,4′,5′-pentahydroxyflavone)), while the treatment downregulated the metabolites mwshy0080 (luteolin-7-O-neohesperidoside (lonicerin)) and mwshy0050 (kaempferol-3-O-rutinoside (nicotiflorin)). The 250 mg/kg Pb treatment upregulated the flavonoid metabolites mwshy0017 (naringenin (5,7,4′-trihydroxyflavanone), Lmzp002365 (hesperetin-7-O-glucoside), mwshy0089 (5,4′-dihydroxy-7-methoxyflavanone (sakuranetin)), mwshy0008 (apigenin-6-C-glucoside (isovitexin)), mwshy0080 (luteolin-7-O- neohesperidoside (lonicerin)), mwshy0050 (kaempferol-3-O-rutinoside (nicotiflorin)), mwshy0136 (kaempferol-3-O-glucoside (astragalin)), mwshy0067 (quercetin-3-O-rutinoside (rutin)), pme1201 (phloretin), pme3227 (vitexin-2″-O-rhamnoside), mws1179 (naringenin-7-O-glucoside (prunin)), mws0178 (chlorogenic acid (3-O-caffeoylquinic acid)), mws0914 (pinobanksin), and mws0048 (apigenin-8-C-glucoside (vitexin)) and downregulated pme0001 (hesperetin-7-O-neohesperidoside (neohesperidin)).

### 2.5. Pathway Enrichment of Flavonoid-Related Genes and Metabolites

**Figure 5:**
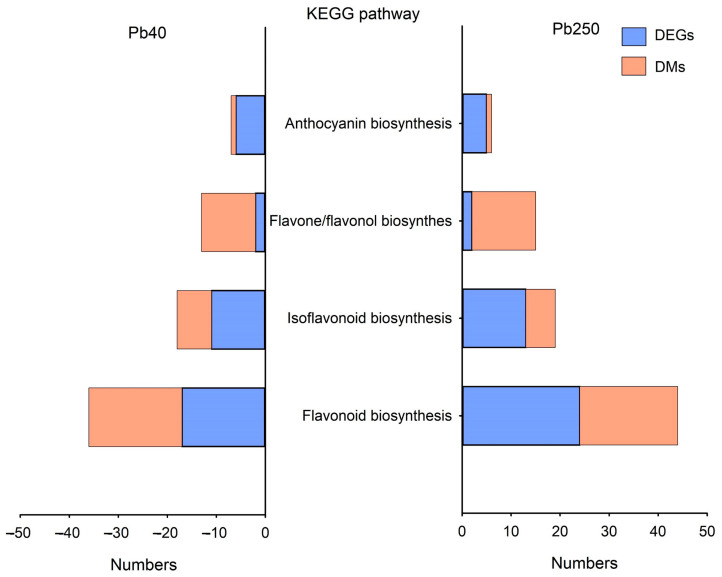
Pb-induced enrichment analysis of differentially expressed gene and metabolite pathways in maize roots. DEGs: differentially expressed genes; DMs: differential metabolites. Pb40 and Pb250 refer to the concentrations of lead at 40 mg/kg and 250 mg/kg, respectively.

According to the enrichment pathway results (Figure 5), the 40 and 250 mg/kg Pb treatments induced significant enrichment of differentially expressed genes and metabolites of flavonoids in maize roots; these were involved in the flavonoid, isoflavonoid, flavone, and anthocyanin biosynthesis pathways. Among these pathways, flavonoid biosynthesis had the highest number of differentially expressed genes and metabolites.

**Figure 6:**
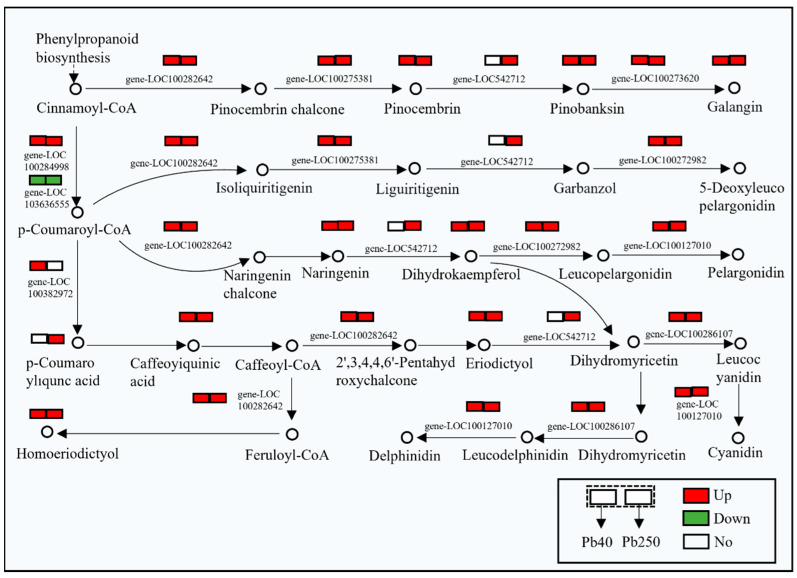
Flavonoid anabolism altered by Pb exposure. Genes and metabolites with significant changes in expression are shown in red (upregulated) or green (downregulated) boxes. Pb40 and Pb250 refer to the concentrations of lead at 40 mg/kg and 250 mg/kg, respectively.

The interaction network of the enrichment pathways (Figure 6) demonstrated that, in the flavonoid biosynthesis pathway, Pb exposure significantly upregulated the genes LOC100282642, LOC100273620, LOC100284998, LOC100284998, LOC100127010, LOC100272982, and LOC100127010 and significantly downregulated LOC103636555. Furthermore, Pb exposure significantly upregulated the metabolites pinocembrin, galangin, caffeoylquinic acid, eriodictyol, and homoeriodictyol. These differentially expressed genes and metabolites were found to be indirectly involved in isoflavonoid biosynthesis, flavone and flavonol biosynthesis, or anthocyanin biosynthesis.

## 3. Discussion

An integrated transcriptomic and metabolomic analysis is useful for identifying the molecular mechanisms underlying Pb regulation of flavonoid biosynthesis in maize root systems. Such an approach has been successfully applied to flavonoid biosynthesis in the sea buckthorn (*Hippophae rhamnoides* L.) root system and the mechanism of cycloheximide impact on Cyclocarya paliurus [35,36]. This study revealed that the phenotypic characteristics of maize roots, including the length and fresh weight, were not significantly altered by exposure to different levels of Pb. Nevertheless, the integrated transcriptomic and metabolomic analysis revealed that different levels of Pb exposure induced differential expression of flavonoid-related genes and metabolites in maize roots. These genes and metabolites were involved in hydroxycinnamoyl transferase activity, flavanone 4-reductase activity, dihydrokaempferol 4-reductase activity, and isoflavone 2′-hydroxylase activity and were significantly enriched in flavonoid biosynthesis and isoflavonoid biosynthesis pathways. The results suggest that Pb exposure interferes with the biosynthesis and metabolic processes of flavonoids in maize roots. Similarly, other studies have found that abiotic stress factors, such as cadmium, cold, drought, and salt, can also affect the biosynthesis and metabolism of plant flavonoids [37,38]. To the best of our knowledge, this study is the first to integrate transcriptomics and metabolomics in order to identify the molecular regulatory mechanisms of flavonoid biosynthesis in maize roots.

Exposure to Pb interferes with the flavonoid biosynthesis pathway, an important source of flavonoids [39]. Previous studies have shown that drought stress upregulated the genes F3H, CHS, FLS, ANS, FNS, DFR, and CHI, leading to increased total flavonoid and anthocyanin contents in wheat leaves [40]. Low temperature and salt stress upregulated the genes DFR, CHS, F3H, AN, and CHI, leading to increased anthocyanin content in Arabidopsis thaliana leaves [41]. These results indicate that abiotic stress can affect flavonoid content by regulating synthase genes in the flavonoid biosynthesis pathway [35]. In the present study, several key genes upstream of the flavonoid biosynthesis pathway in maize roots (e.g., LOC100282642, LOC100273620, LOC100284998, LOC100272982, and LOC100127010) were upregulated by Pb stress, while multiple downstream flavonoid metabolites, such as pinocembrin, galangin, caffeoylquinic acid, eriodictyol, and homoeriodictyol, were also upregulated. These results suggest that Pb exposure is likely to increase plant flavonoid content by regulating the expression of genes in the flavonoid biosynthesis pathway in maize roots.

Pb exposure may increase the content of flavonoids, thereby enhancing the lead tolerance of maize roots. Due to their ability to bind to heavy metals, flavonoids have received significant attention concerning plant tolerance to heavy metal stress. Flavonoids can bind to heavy metals and transport them to vesicles for compartmentalization, thereby reducing the intracellular levels of free metals and mitigating the toxic effects of heavy metals on plants [28]. Cadmium (Cd) increased the content of anthocyanins on the surface of rice leaves, promoting the vacuolar segregation of Cd elements and thereby reducing the Cd content and ultimately improving the Cd tolerance of rice plants [27]. Other studies have found that boron and arsenic can increase the contents of flavonoids and anthocyanins in plant leaves, thereby increasing plant tolerance to metalloids [24,25,26]. Moreover, this study found that lead could significantly increase the content of flavonoids; this may be a compensatory mechanism for the enhanced tolerance of maize roots to Pb.

In this study, high concentrations of Pb exposure resulted in significant accumulation of Pb in maize roots but did not significantly alter the growth of the maize root system. The possible cause of this phenomenon may be related to flavonoid accumulation. Flavonoids play significant roles in plant antioxidant damage, growth, and development due to the hydroxyl group of the catechol group on the B-ring of flavonoids that can directly bind to ROS [42,43]. This work has demonstrated that exposure to high concentrations of Pb significantly upregulated the content of flavonoids in maize roots, but there were no significant changes in root growth. The results suggest that Pb is likely to maintain the normal growth of maize roots by increasing the content of flavonoids and reducing oxidative damage [44,45].

## 4. Materials and Methods

### 4.1. Experimental Design

Maize cultivar B was obtained from Lanping County, Yunnan Province, China. Maize seeds were cultivated in three treatment groups, a control group (without added Pb), 40 mg/kg of Pb treatment group, and 250 mg/kg of Pb treatment group. Each treatment had three parallel replicates. Maize cultivation conditions included a soil:sand mixture of 1:1 (*w*/*w*), with temperature and humidity maintained at 16–24 °C and 60–80%, respectively. Fourteen days after outdoor planting, the roots of maize plants were sampled. After rapid freezing with liquid nitrogen, the samples were stored at −80 °C until use [20]. At 20 days after outdoor planting, maize seedlings were selected and washed with water, and the fresh weights of the above- and below-ground parts were measured. The seedlings were then dried at 65 °C and again weighed. A total of twenty-seven pots were planted, one per treatment, with six maize plants in each pot. The pots measured 52 cm in width and 34 cm in height.

### 4.2. Determination of the Pb Concentration in Maize Roots

The Pb concentration was determined following Gupta et al. [46] for sample pretreatment. Briefly, 0.1 g of maize roots (dry weight) was added to 12 mL of aqua regia (i.e., nitric acid/hydrochloric acid (3:1)) in a 100 mL conical flask. After being soaked overnight, the samples were digested on a hot plate. If the samples turned a brownish-black color during the heating process, more nitric acid was added until white smoke was generated; however, the digested solution was colorless or yellowish. When approximately 1 mL of solution remained in the flasks, the flasks were removed from the hot plate and cooled. The digested solution was then transferred to a 25 mL volumetric flask. Prior to this, each triangular flask had been washed three times with deionized water. The washing solution was placed in a measuring flask, diluted to the scale line with deionized water, and mixed well. Flame atomic absorption spectrometry was performed to determine the Pb content.

### 4.3. Characterization of Maize Root Morphology

The phenotypes and growth indices of maize roots were determined. For each treatment, three seedlings were selected, cleaned with tap water, brought back to the laboratory, and photographed against a black cloth background using an α7R IV camera (Sony, Tokyo, Japan). Furthermore, the 20-day maize seedlings were used for determining growth index statistics. The soil was washed off the maize roots with deionized water, and then the seedlings were washed and drained on filter paper to separate the roots from the above-ground parts. Three seedlings were randomly selected as replicates, and their fresh mass (FM) and root length (RL) were determined. Sets of three biological replicates were used.

### 4.4. Transcriptomic Analysis of Maize Roots

Roots from the control and Pb-treated maize seedlings were collected. Total RNA was extracted using an RNeasy Micro Kit (Qiagen, Hilden, Germany). The cDNA was analyzed using an Illumina sequencer (Illumina, San Diego, CA, USA), and the cDNA library was constructed by Metware Biotechnology Co., Ltd. (Wuhan, China). Clean reads were aligned with the maizeGDB B73 genome (https://maizegdb.org (accessed on 21 September 2023)). Data analysis and mapping were parsed by the free platform Metware Cloud (https://cloud.metware.cn (accessed on 21 September 2023)). Principal component analysis and cluster heatmap analysis were conducted using R version 3.5.1 and R version 4.2.0, respectively.

### 4.5. Metabolomic Analysis of Maize Roots

Roots were collected from control and Pb-treated maize seedlings. After vacuum freeze-drying, the samples were ground at 30 Hz for 1 min and 30 s. The extraction and preparation of the samples prior to ultra-performance liquid chromatography–mass spectrometry (UPLC-MS) were performed according to Chen et al. [47]. The instruments needed for UPLC and electrospray ionization–quadrupole linear ion trap mass spectrometry were supplied by Wuhan Metware Biotechnology Co., Ltd., Wuhan, China. The data were analyzed and plotted using the free platform Metware Cloud (https://cloud.metware.cn (accessed on 21 September 2023)). The software of principal component analysis and cluster heatmap analysis was consistent with transcriptomics analysis. KEGG pathways were mapped using the OmicStudioKits (v1.8.1) online tool (https://www.omicstudio.cn/tool (accessed on 21 September 2023)).

### 4.6. Validation of Gene Expression

In order to verify the reliability of transcriptional level data, the expression levels of multiple genes (LOC100286107, LOC100275381, LOC100285776, LOC542712, LOC100192114) were measured through quantitative reverse transcription polymerase chain reaction (qRT-PCR). The RNA samples used were the same as those used for sequencing. cDNA was synthesized using a BioRT Master HiSensi cDNA First Strand Synthesis kit and then analyzed using a LightCycle 96sw qPCR instrument (Roche, Basel, Switzerland). The actin gene of maize was used as the internal reference gene for the normalization of the expression of selected genes. The expression of genes was analyzed by the 2^−∆∆CT^ method [48,49]. The primers for the genes are listed in Table S1.

### 4.7. Statistical Analysis

The omics data were analyzed using the Metware Cloud, a free online platform for data analysis (https://cloud.metware.cn (accessed on 21 September 2023)). The other data were represented as mean ± standard deviation. The Kolmogorov–Smirnov test was employed to test for normality and homogeneity of variance of Pb content data in maize roots. SPSS 19.0 was used for one-way ANOVA, and multiple comparisons were performed by the Duncan test. A value of *p* < 0.05 was considered statistically significant.

## 5. Conclusions

This is the first report analyzing the molecular mechanism of flavonoid biosynthesis in maize roots under Pb stress through integrating metabolomic and transcriptomic methods. The results of multi-omics confirmed that exposure to Pb can lead to the upregulation of genes and metabolites related to flavonoids in maize roots, in turn affecting the synthesis of flavonoids. The pathways of flavonoid, isoflavonoid, and flavonol biosynthesis are key pathways influenced by Pb. This study provides strong evidence that Pb exposure can increase the levels of flavonoids and thus lead to increased tolerance of maize roots to Pb stress. The results of this study provide a new perspective for understanding the mechanism of the impact of lead on maize roots. Nevertheless, this study is still limited. In order to validate the results of this study across different locations and maize varieties, future research should use multiple maize varieties to investigate the changes in flavonoids under lead exposure in different time periods and locations.

## Figures and Tables

**Table 1:** Validation of gene expression after Pb exposure in maize roots.

Gene ID	Control	Pb40	Pb250
LOC100286107	0.30 ± 0.10	0.31 ± 0.09 *	5.54 ± 0.44 **
LOC100275381	1.19 ± 0.05	3.94 ± 2.12 **	0.70 ± 0.03 **
LOC100285776	0.78 ± 0.27	0.69 ± 0.32	5.27 ± 3.02 **
LOC542712	17.39 ± 0.52	20.24 ± 1.39	5.0 ± 2.17 **
LOC100192114	0.07 ± 0.01	0.22 ± 0.10 *	1.23 ± 0.08 **

Note: ** Represents a highly significant difference in the Pb treatment group compared to the control group, *p* < 0.01; * represents a significant difference in the Pb treatment group compared to the control group, *p* < 0.05. Pb40 and Pb250 refer to the concentrations of lead at 40 mg/kg and 250 mg/kg, respectively.

**Table 2:** Gene-Ontology-based functional analyses of the flavonoid-related differentially expressed genes following Pb exposure in maize roots ^#^.

		Pb40	Pb250
GO_ID	GO_Description	Number of Enriched Genes	Number of Enriched Genes
GO:0009813	flavonoid biosynthetic process	12	12
GO:0016758	transferase activity	9	11
GO:0005506	iron ion binding	12	11
GO:0008152	metabolic process	8	10
GO:0009718	anthocyanin-containing compound biosynthetic process	9	10
GO:0046872	metal ion binding	7	7
GO:0050734	hydroxycinnamoyltransferase activity	4	7
GO:0016021	integral component of membrane	7	7
GO:0031418	L-ascorbic acid binding	4	4
GO:0050662	coenzyme binding	4	4
GO:0010023	proanthocyanidin biosynthetic process	3	3
GO:0045430	chalcone isomerase activity	3	3
GO:0050589	leucocyanidin oxygenase activity	3	3
GO:0045552	dihydrokaempferol 4-reductase activity	3	3
GO:0047890	flavanone 4-reductase activity	3	3
GO:0009753	response to jasmonic acid	3	3
GO:0033773	isoflavone 2′-hydroxylase activity	4	3
GO:0016210	naringenin–chalcone synthase activity	2	2
GO:0016705	oxidoreductase activity	2	2
GO:0097237	cellular response to toxic substance	2	2

^#^ Pb40 and Pb250 refer to the concentrations of lead at 40 mg/kg and 250 mg/kg, respectively.

## Data Availability

Data will be made available on request.

## Supplementary Materials

The following supporting information can be downloaded at: https://www.mdpi.com/article/10.3390/ijms25116050/s1.
