# Correction: Guo et al. Integrated Transcriptomic and Metabolomic Analysis Reveals the Molecular Regulatory Mechanism of Flavonoid Biosynthesis in Maize Roots Under Lead Stress. *Int. J. Mol. Sci.* 2024, *25*, 6050

**DOI:** 10.3390/ijms27125415

**Published:** 2026-06-16

**Authors:** Zhaolai Guo, Xinqi Yuan, Ting Li, Sichen Wang, Yadong Yu, Chang’e Liu, Changqun Duan

**Affiliations:** 1 Yunnan Key Laboratory of Plateau Ecology and Degraded Environment Restoration, School of Ecology and Environmental Science, Yunnan University, Kunming 650091, China; 18314522882@163.com (Z.G.); xqyuan2022@163.com (X.Y.); litingkm1891@163.com (T.L.); wangsichen0101@163.com (S.W.); 13685311866@163.com (Y.Y.); change@ynu.edu.cn (C.L.); 2 Yunnan Provincial Innovative Research Team of Environmental Pollution, Food Safety, and Human Health, Institute of Environmental Remediation and Human Health, School of Ecology and Environment, Southwest Forestry University, Kunming 650224, China

## Supplementary Materials

The authors realized that the Supplementary Materials are missing in the original published version of [1] “Integrated Transcriptomic and Metabolomic Analysis Reveals the Molecular Regulatory Mechanism of Flavonoid Biosynthesis in Maize Roots under Lead Stress”. Therefore, the authors have re-provided the Supplementary Materials and hope to update them by publishing a correction notice. The authors state that the scientific conclusions are unaffected. This correction was approved by the Academic Editor. The original publication has also been updated.
