# Supplementary material for: Integrated Transcriptomic and Metabolomic Analysis Reveals the Molecular Regulatory Mechanism of Flavonoid Biosynthesis in Maize Roots under Lead Stress
Source: Int J Mol Sci. 2024 May 31;25(11):6050. doi: 10.3390/ijms25116050 (PMC13285848; doi:10.3390/ijms25116050)
Supplement: Supplementary file 1 [file ijms-25-06050-s001.zip › ijms-2999833-supplementary.pdf]

Supporting information

Table S1. Primer sequences for validating differentially expressed genes

Gene ID	Sequence	Product length
gene-LOC100286107	F- TGTCCAAAGACCCTGAGA	110
	R- GGAAGTGAAGACGATGCG	
gene-LOC100275381	F- GGGAAAGTGGCGGAGAAC	140
	R- TGCGTGAAGTGGATGGAG	
gene-LOC100285776	F- TTCAACTACTACCCTCCC	206
	R- CCATTGTTCATTATCTCC	
gene-LOC542712	F- GGTGGTGAACTCGGAGTG	128
	R- CGAAGGTGATGGGATGGT	
gene-LOC100192114	F- GAGGGTGTCGGGGAGAGG	94
	R- GGCGTGGGTGAGGTAGCA	
Actin-maize	F-CCAAGGCCAACAGAGAGAAA	210
	R-CCAAACGGAGAATAGCATGAG	

Table S2. Expression of genes involved in flavonoid synthesis under Pb40mg/kg stress

ID	log2FoldChange	pvalue	regulated	gene_description
gene-LOC103636555	-3.09523	3.2E-169	down	--
gene-LOC100284998	2.154694	5.9E-119	up	trans-cinnamate 4-monooxygenase
gene-LOC100275381	2.036827	1.62E-71	up	uncharacterized LOC100275381
gene-LOC100279536	-1.68364	2.83E-71	down	putative cytochrome P450 superfamily protein
gene-LOC100284770	1.645988	3.14E-43	up	uncharacterized LOC100284770
gene-LOC100304381	-1.34885	3.45E-39	down	UDP-glycosyltransferase 88A1
gene-LOC100382629	-1.2737	2.83E-38	down	uncharacterized LOC100382629
gene-LOC100282642	1.859788	8.63E-34	up	chalcone synthase
gene-LOC100273620	1.487127	1.2E-32	up	jasmonate-regulated gene 21
gene-LOC103655055	2.291253	7.7E-27	up	--
gene-LOC100284018	0.884178	1.18E-26	false	chalcone--flavonone isomerase
gene-LOC100191664	-1.0231	3.24E-26	down	UDP-glycosyltransferase 88A1
gene-LOC100272982	1.762584	9.61E-26	up	anthocyaninless 4
gene-LOC100274392	-1.1094	9.76E-26	down	uncharacterized LOC100274392
gene-LOC100273344	4.979595	9.22E-23	up	putative cytochrome P450 superfamily protein
gene-LOC100276821	0.990672	1.5E-21	false	chalcone flavanone isomerase 1
gene-LOC100382972	1.822825	9.28E-21	up	Spermidine hydroxycinnamoyl transferase
gene-LOC100274473	-1.15017	3.23E-20	down	putative cytochrome P450 superfamily protein
gene-LOC103630531	-2.08595	5.41E-17	down	--
gene-LOC100274415	0.724827	2.63E-13	false	chalcone synthase
gene-LOC100286107	2.308722	8.75E-12	up	uncharacterized LOC100286107
gene-LOC103638801	1.200755	1.14E-08	up	--

gene-LOC103642652	1.041547	6.88E-08	up	--
gene-LOC109944638	1.298156	8.2E-08	up	--
gene-LOC103626938	-1.78545	1E-07	down	--
gene-LOC103632694	-1.51565	8.88E-07	down	--
gene-LOC103639274	3.339408	2.07E-06	up	--
gene-LOC103625868	3.697831	3.08E-06	up	--
gene-LOC100279846	-1.06253	3.42E-06	down	UDP-glycosyltransferase 88A1
gene-LOC118474966	-2.94652	3.6E-06	down	--
gene-LOC103649816	1.362222	6.83E-06	up	--
gene-LOC103640406	0.663307	9.53E-06	false	--
gene-LOC103633203	1.749425	9.97E-06	up	--
gene-LOC103652790	2.148328	1.95E-05	up	--
gene-LOC100283088	0.283921	0.000272	false	anthocyanidin 5,3-O-glucosyltransferase
gene-LOC542712	-0.70411	0.000418	false	uncharacterized LOC542712
gene-LOC103646891	-1.15273	0.001428	down	--
gene-LOC100127010	1.36717	0.005142	up	anthocyaninless 2
gene-LOC103647933	1.327976	0.016866	up	--
gene-LOC100192114	0.390304	0.133754	false	Anthocyanidin 3-O-glucoside 2'''-O-xylosyltransferase
gene-LOC113839550	0.33571	0.275139	false	uncharacterized LOC113839550
gene-LOC100285776	0.171505	0.350857	false	leucoanthocyanidin dioxygenase
novel.6134	0.140254	0.422728	false	--
novel.3479	0.210749	0.430054	false	--
gene-LOC100284504	0.230447	0.522248	false	dihydroflavonol-4-reductase
gene-LOC103633102	0.24466	0.607335	false	--
gene-LOC100274555	0.069221	0.655811	false	uncharacterized LOC100274555

Table S3. Expression of genes involved in flavonoid synthesis under Pb250mg/kg stress

ID	log2FoldChange	pvalue	regulated	gene_description
gene-LOC100282642	3.952123104	5.6917E-221	up	chalcone synthase
gene-LOC100284998	2.879178401	1.2843E-210	up	trans-cinnamate 4-monooxygenase
gene-LOC103640406	3.119496565	2.4943E-133	up	--
gene-LOC100274415	2.26739049	4.1752E-112	up	chalcone synthase
gene-LOC100272982	2.763934957	2.38092E-69	up	anthocyaninless 4
gene-LOC100284018	1.845380218	3.65553E-58	up	chalcone--flavonone isomerase
gene-LOC109944638	2.77040811	2.94939E-40	up	--
gene-LOC100274555	1.448934966	3.55246E-28	up	uncharacterized LOC100274555
gene-LOC100283088	1.02332659	6.95519E-26	up	anthocyanidin 5,3-O-glucosyltransferase
gene-LOC103636555	-1.21953423	2.37711E-24	down	--
gene-LOC100276821	1.099717282	1.0481E-20	up	chalcone flavanone isomerase 1
gene-LOC100284504	2.045974101	4.86443E-20	up	dihydroflavonol-4-reductase
gene-LOC103633102	2.844581704	3.81009E-18	up	--
gene-LOC100275381	1.165565646	1.52946E-16	up	uncharacterized LOC100275381
gene-LOC103642652	1.548306527	2.06841E-16	up	--
gene-LOC100273620	1.140317032	1.40613E-14	up	jasmonate-regulated gene 21

gene-LOC113839550	1.829187108	7.83649E-14	up	uncharacterized LOC113839550
gene-LOC542712	1.24861561	8.57471E-13	up	uncharacterized LOC542712
gene-LOC103633813	4.063960403	7.16264E-12	up	--
gene-LOC103649821	3.26079368	3.56558E-11	up	--
gene-LOC100286107	2.297276244	3.75741E-11	up	uncharacterized LOC100286107
gene-LOC100279536	0.583049685	8.80879E-11	false	putative cytochrome P450 superfamily protein
gene-LOC100127010	2.931750103	3.52104E-10	up	anthocyaninless 2
gene-LOC103648690	3.281443899	1.35132E-09	up	--
novel.6134	-1.28775737	9.26197E-09	down	--
gene-LOC103638801	1.036011383	7.11486E-08	up	--
gene-LOC103639274	3.617904623	1.60045E-07	up	--
gene-LOC103647631	2.615971208	9.68235E-07	up	--
gene-LOC103649816	1.476248402	3.50051E-06	up	--
gene-LOC100382629	-0.46520235	6.06133E-06	false	uncharacterized LOC100382629
gene-LOC100281894	4.720143109	9.12727E-06	up	transferase
gene-LOC100273344	3.025806012	1.58978E-05	up	putative cytochrome P450 superfamily protein
novel.3479	1.136492973	4.67127E-05	up	--
gene-LOC103632694	-1.06132	0.000257359	down	--
gene-LOC103652790	1.766057533	0.001731044	up	--
gene-LOC100284770	-0.34974054	0.003160078	false	uncharacterized LOC100284770
gene-LOC100382972	0.688168103	0.004940123	false	Spermidine hydroxycinnamoyl transferase
gene-LOC100192114	-1.00687534	0.00613526	down	Anthocyanidin 3-O-glucoside 2'''-O-xylosyltransferase
gene-LOC103646891	-0.92554679	0.01095441	false	--
gene-LOC100285776	0.394239144	0.014958317	false	leucoanthocyanidin dioxygenase
gene-LOC103655055	0.699745905	0.019499933	false	--
gene-LOC103635745	1.334757568	0.020163606	up	--
gene-LOC100274392	0.262889536	0.035025415	false	uncharacterized LOC100274392
gene-LOC103626938	-0.49810355	0.065119663	false	--
gene-LOC100191664	-0.17413448	0.068050724	false	UDP-glycosyltransferase 88A1
gene-LOC103647933	0.942065404	0.102657607	false	--
gene-LOC100274473	-0.15586021	0.104329643	false	putative cytochrome P450 superfamily protein
gene-LOC118474966	-1.08699939	0.132299032	false	--
gene-LOC100279846	-0.2534518	0.255924475	false	UDP-glycosyltransferase 88A1
gene-LOC103633203	0.519963999	0.351121275	false	--
gene-LOC100304381	-0.09894021	0.3784767	false	UDP-glycosyltransferase 88A1
gene-LOC103630531	0.070953323	0.766256306	false	--

Table S4. Expression of related metabolites in the flavonoid synthesisunder Pb40mg/kg stress

Index	Compounds	VIP	p_value	FDR	Log2FC	Type
mws0789	Pinocembrin (Dihydrochrysin)	1.189221	0.001979	0.008618	14.45965	up
MWS20151	Apigenin	1.18324	0.00036	0.00425	4.35448	up

		3	7	1	6	
Lmyn006227	Galangin (3,5,7-Trihydroxyflavone)	1.179789	8.82E-06	0.000946	3.76586	up
MWSHY0017	Naringenin (5,7,4'-Trihydroxyflavanone)	1.189025	0.001257	0.00704	5.451221	up
mws0914	Pinobanksin	1.187444	0.000697	0.005513	1.471239	up
pme1201	Phloretin	1.166602	0.008818	0.021955	3.074586	up
pme3233	Calycosin	1.183689	0.0017	0.00807	3.564702	up
mws0051	Acacetin	1.187718	0.000884	0.006043	6.938788	up
mws0918	Prunetin (5,4'-Dihydroxy-7-methoxyisoflavone)	1.186983	4.66E-05	0.001659	6.911364	up
pme0088	Luteolin (5,7,3',4'-Tetrahydroxyflavone)	1.18627	0.000468	0.004714	2.934562	up
MWSHY0089	5,4'-Dihydroxy-7-methoxyflavanone (Sakuranetin)*	1.180997	0.000531	0.004918	9.729301	up
mws1094	Aromadendrin (Dihydrokaempferol)	1.178559	0.002698	0.010486	5.538965	up
Lmdn006025	2-Hydroxy-2,3-dihydrogenistein	1.188942	0.000741	0.005513	3.946357	up
mws0064	Eriodictyol (5,7,3',4'-Tetrahydroxyflavanone)	1.185784	0.000722	0.005513	3.708424	up
mws0920	Tricetin (5,7,3',4',5'-Pentahydroxyflavone)	1.159177	0.027768	0.050139	3.464803	up
mws1033	Homoeriodictyol	1.189129	0.005394	0.015497	12.86648	up
MWSHY0049	Hesperetin	1.182263	0.002751	0.01057	3.244525	up
mws0044	Dihydroquercetin(Taxifolin)	1.186497	0.000528	0.004918	2.61796	up
pmb3074	5-O-p-Coumaroylquinic acid	1.174402	0.000692	0.005513	-0.65918	insig
mws0178	Chlorogenic acid (3-O-Caffeoylquinic acid)	1.161627	0.004079	0.013038	1.25656	up
Lmfn005487	Quercetin-3-O-Sulfonate	1.174388	0.002276	0.009338	2.62071	up
pme3504	Formononetin-7-O-glycoside (Ononin)	1.173749	0.002229	0.009297	4.887171	up
MWSHY0189	Apigenin-7-O-glucoside(Cosmosiin)	1.182432	0.008645	0.021657	2.532279	up
MWSHY00	Apigenin-6-C-glucoside (Isovitexin)	0.60539	0.35659	0.41777	0.32317	insig

08		3	4	2	1	
mws0048	Apigenin-8-C-Glucoside (Vitexin)	0.960647	0.054862	0.087656	0.379751	insig
mws1179	Naringenin-7-O-glucoside (Prunin)	1.175867	0.005097	0.014858	3.253191	up
Lmlp006175	Isosalipurposide (Phlorizin Chalcone)	1.170757	0.001483	0.00775	1.189997	up
MWSHY0136	Kaempferol-3-O-glucoside (Astragalin)	1.121191	0.00162	0.007881	-1.44669	down
mws0091	Quercetin-3-O-glucoside (Isoquercitrin)	1.150917	0.025451	0.046731	-1.71266	down
Lmzp002365	Hesperetin-7-O-glucoside	1.163833	0.018741	0.037205	2.1682	up
pmp000194	6"-O-Malonylgenistin	1.186995	0.001436	0.007665	3.732024	up
pmb0542	Cyanidin-3-O-(6"-O-malonyl)glucoside	1.131357	0.016482	0.034092	-1.23832	down
pme3227	Vitexin-2"-O-rhamnoside	1.147538	0.016011	0.033374	-1.00016	down
MWSHY0050	Kaempferol-3-O-rutinoside(Nicotiflorin)	1.157449	0.01877	0.037205	-2.1841	down
MWSHY0080	Luteolin-7-O-neohesperidoside (Lonicerin)	1.181721	0.00583	0.016327	-2.14143	down
MWSHY0067	Quercetin-3-O-rutinoside (Rutin)	1.077925	0.194114	0.251667	-2.80465	down
pme0001	Hesperetin-7-O-neohesperidoside(Neohesperidin)	1.156368	0.002047	0.008755	1.082614	up

Table S5. Expression of related metabolites in the flavonoid synthesis under Pb250mg/kg stress

Index	Compounds	VIP	p_value	FDR	Log2FC	Type
mws0789	Pinocembrin (Dihydrochrysin)	1.180225	0.000458	0.004157	14.64166	up
MWS20151	Apigenin	1.179056	0.02379	0.043404	-9.83923	down
Lmyn006227	Galangin (3,5,7-Trihydroxyflavone)	1.171924	0.000271	0.003563	4.025591	up
MWSHY0017	Naringenin (5,7,4'-Trihydroxyflavanone)	1.180197	6.78E-07	0.000338	6.43888	up
mws0914	Pinobanksin	1.176355	0.010032	0.02335	3.464736	up
pme1201	Phloretin	1.171713	0.002716	0.009964	4.646758	up
mws0051	Acacetin	1.178763	0.000619	0.00481	7.126509	up
mws0918	Prunetin (5,4'-Dihydroxy-7-methoxyisoflavone)	1.178025	0.000141	0.002599	7.065564	up
pme0088	Luteolin (5,7,3',4'-Tetrahydroxyflavone)	1.163373	0.016899	0.033866	2.279559	up
MWSHY0089	5,4'-Dihydroxy-7-methoxyflavanone (Sakuranetin)*	1.17414	0.001158	0.006531	11.54781	up
mws1094	Aromadendrin (Dihydrokaempferol)	1.167937	0.00211	0.008643	5.142416	up
Lmdn006025	2-Hydroxy-2,3-dihydrogenistein	1.180034	0.002018	0.008427	7.31598	up

mws0064	Eriodictyol (5,7,3',4'-Tetrahydroxyflavanone)	1.173994	0.00226	0.009024	2.965541	up
mws0920	Tricetin (5,7,3',4',5'-Pentahydroxyflavone)	1.137099	0.001306	0.006895	2.325418	up
mws1033	Homoeriodictyol	1.180191	0.001131	0.006413	11.59776	up
MWSHY0049	Hesperetin	1.172232	0.000498	0.004336	2.814313	up
mws0044	Dihydroquercetin(Taxifolin)	1.17756	0.00134	0.007004	2.810601	up
pmb3074	5-O-p-Coumaroylquinic acid	1.177096	0.011655	0.025734	4.401457	up
mws0178	Chlorogenic acid (3-O-Caffeoylquinic acid)	1.177108	0.000162	0.002746	2.941887	up
Lmfn005487	Quercetin-3-O-Sulfonate	1.163202	0.000571	0.004597	2.172653	up
pme3504	Formononetin-7-O-glycoside (Ononin)	1.092459	0.029258	0.050897	2.235118	up
MWSHY0189	Apigenin-7-O-glucoside(Cosmosiin)	1.179747	9.35E-05	0.002195	4.537351	up
MWSHY0008	Apigenin-6-C-glucoside (Isovitexin)	1.175552	8.8E-06	0.000798	1.631077	up
mws0048	Apigenin-8-C-Glucoside (Vitexin)	1.157496	0.00251	0.009481	1.532351	up
mws1179	Naringenin-7-O-glucoside (Prunin)	1.176986	4.26E-05	0.00177	6.069413	up
Lmlp006175	Isosalipurposide (Phlorizin Chalcone)	1.176799	0.002039	0.008479	2.765498	up
MWSHY0136	Kaempferol-3-O-glucoside (Astragalin)	1.177533	0.002905	0.010318	2.77417	up
mws0091	Quercetin-3-O-glucoside (Isoquercitrin)	1.177071	0.003521	0.011577	5.531291	up
Lmzp002365	Hesperetin-7-O-glucoside	1.1706	0.004509	0.013566	2.79134	up
pmp000194	6"-O-Malonylgenistin	1.178138	0.000777	0.005527	3.731471	up
pmb0542	Cyanidin-3-O-(6"-O-malonyl)glucoside	1.176628	0.006304	0.017143	4.683052	up
pme3227	Vitexin-2"-O-rhamnoside	1.171729	3.52E-05	0.001597	1.960645	up
MWSHY0050	Kaempferol-3-O-rutinoside(Nicotiflorin)	1.131801	0.001438	0.007302	1.223893	up
MWSHY0080	Luteolin-7-O-neohesperidoside (Lonicerin)	1.165764	0.000519	0.004387	1.413048	up
MWSHY0067	Quercetin-3-O-rutinoside (Rutin)	1.111468	0.007019	0.018344	3.173751	up
